# Exploring macrophage polarization: biological insights, key laboratory techniques and research perspectives

**DOI:** 10.3389/fimmu.2026.1837287

**Published:** 2026-07-14

**Authors:** Enkhbolor Battumur, John R. Clegg, Handan Acar

**Affiliations:** 1 Stephenson School of Biomedical Engineering, University of Oklahoma, Norman, OK, United States; 2 Stephenson Cancer Center, The University of Oklahoma Health Campus, Oklahoma City, OK, United States; 3 Harold Hamm Diabetes Center, The University of Oklahoma Health Campus, Oklahoma City, OK, United States; 4 Department of Neurosurgery, The University of Oklahoma Health Campus, Oklahoma City, OK, United States; 5 Materials Science and Engineering Program, University of Oklahoma, Norman, OK, United States

**Keywords:** innate immunity, M1/M2 activation, macrophage plasticity, macrophage polarization, phenotypes switch

## Abstract

Macrophages are key cells of the innate immune system and serve as a first line of defense against invading pathogens while maintaining tissue homeostasis. As highly specialized phagocytic cells, they eliminate pathogens, clear apoptotic and abnormal cells, and coordinate both innate and adaptive immune responses. Under steady state conditions, macrophages remain in a quiescent yet surveillance active state, continuously sensing their microenvironment to preserve tissue integrity without initiating unnecessary inflammatory responses. Tissue resident macrophages, such as microglia in the brain and Kupffer cells in the liver, exhibit functional specialization shaped by local environmental cues, enabling organ specific roles adapted to tissue requirements. Recent advances have improved our understanding of molecular and signaling mechanisms underlying macrophage phenotypic diversity and plasticity. However, macrophage biology remains highly complex due to dynamic responses to temporally and spatially variable microenvironmental signals, as well as the co-existence of heterogeneous pro inflammatory and anti-inflammatory states. In addition, experimental challenges persist, including variability in isolation procedures, difficulties in distinguishing overlapping activation states, lack of universally reliable phenotypic markers, and context-dependent functional variability. Furthermore, discrepancies arising from *in vitro* culture systems, animal models, and technical limitations in high dimensional analyses complicate data interpretation and cross study comparisons. Therefore, the development and implementation of standardized and well-optimized experimental protocols including cell isolation, culture conditions, stimulation strategies, multiparametric phenotyping, and complementary functional assays are essential to improve reproducibility and translational relevance. This review summarizes current knowledge on macrophage biology and phenotypic plasticity, highlighting key regulatory mechanisms, experimental limitations, and methodological considerations. A deeper understanding of macrophage behavior through rigorous and standardized approaches will support the development of more effective strategies for targeting these cells in the treatment of autoimmune diseases, infections, cancer, and other chronic inflammatory disorders.

## 1 Introduction to macrophages and their role in the immune system

The innate immune response represents the body’s first line of defense, employing various protective mechanisms including physical and chemical barriers, and also cellular activity that responds quickly and non-specifically to invading pathogens (1, 2). Macrophages are crucial to this process as specialized phagocytic cells which serve as an early line of defense. They are part of the myeloid lineage, arising within the broader hematopoietic system that give rise to innate immune cells such as monocytes, granulocytes, and certain dendritic cell subsets (3). They arise from both embryonic progenitors that seed tissues and self-maintain resident populations, as well as from circulating monocytes that are recruited during inflammation. Rather than being defined by their origin, macrophages are characterized by their function as “big eaters, ” performing phagocytosis, regulating immune responses, and maintaining tissue homeostasis (4). They play a central role in host defense by eliminating pathogens and abnormal cells, including cancer cells, contributing to both innate and adaptive immunity through the clearance of dead cells (5). In addition, macrophages produce cytokines, present antigens (to varying degrees), regulate inflammation, and promote tissue repair (6, 7). In the absence of danger, macrophages are maintained in an inactive state performing activities such as apoptotic cell clearance and maintaining tissue homeostasis without an inflammatory response. While in this resting state, macrophages actively monitor their environment through phagocytosis and receptor-mediated sensing, remaining functionally active and poised to respond to danger signals arising from infection or tissue injury. Also, they are located throughout tissues (lungs, liver, brain, skin etc.) and are adapted to perform differently depending on the tissues or local spatiotemporal signals in their microenvironment. Microglia, for example, are brain-resident macrophages that carry out synapse refinement work while also providing neuronal support and limited and directed responses to injury to minimize collateral damage to successful repair processes. Kupffer cells are liver-resident macrophages that are uniquely adapted to constantly encounter gut-derived products and tolerates non-pathogenic microbiota antigens while clearing pathogenic bacteria, dead cells, and other debris (8). Such specialization leads to varying expression and activity of macrophage gene expression and surface receptors respectively, which otherwise all macrophages possess the same capacity for rapid and effective responses to signals of danger throughout the body. In recent decades, significant advances have been made toward understanding the mechanisms of driving changes in macrophage phenotypes. However, macrophage behavior remains complex and incompletely understood due to their dynamic phenotypic heterogeneity and the wide range of microenvironmental signaling pathways that influence their phenotype aspects (7, 9). The complexity of macrophage biology presents several significant challenges, including the difficulty in distinguishing heterogeneous macrophage populations displaying pro- or anti-inflammatory phenotypes, identifying bona fide and phenotypically reliable biomarkers, and the complications arising from technological limitations in accurately analyzing and interpreting their cellular behavior in macrophage biology research. Thus, elucidating how macrophages change their phenotype in different environments is crucial for developing innovative strategies to regulate their pro- or anti-inflammatory properties and to enhance the precision and effectiveness of therapeutic and diagnostic applications across a wide range of conditions, including autoimmune disorders, infectious diseases, cancer, and chronic inflammatory diseases (10, 11). Importantly, this functional diversity of macrophages does not resolve into well-defined, fixed categories; rather, the activation states of macrophage co-exist and continuously shaped by the local microenvironmental signals.

### 1.1 Overview of macrophage polarization and classification

Macrophages demonstrate considerable functional versatility, enabling their phenotypic transition from a resting state to being activated and to vary their function based on the tissue environments in which they originate (7). For the purposes of experimental discussion and clarity, this review uses the M0, M1, and M2 framework as a conceptual scaffold, while acknowledging that *in vivo* macrophage phenotypes are far more heterogeneous and dynamic. Under homeostatic conditions, macrophages function at rest (termed as M0 macrophages), perform homeostatic processes such as monitoring tissue integrity, or clearing apoptotic and senescent cells as explained previously. During periods of injury or exposure to pathogens, they detect danger signals via their pattern recognition receptors (PRRs), and present a foreign antigen, and subsequently initiate and shape adaptive immune responses (12, 13). In response to these signals, macrophages develop different functional states based on the local microenvironment. This process is referred to as “macrophage polarization” since the macrophage does produce multiple functional phenotypes across the pro-inflammatory and anti-inflammatory spectrum in response to environmental signal (14, 15). Once the inflammatory signal, or injury signal ceases, the macrophage will return to the resting, M0 type state for tissue homeostasis. This is a highly plastic and flexible process allowing macrophages to assess the complex environmental signals, translocate if necessary, and help maintain the balance between inflammation and tissue health. Given this high plasticity and ability of activation states to co-exist, macrophage polarization is broadly categorized into M1 and M2 types as conceptual reference points rather than discrete biological states, based on functional phenotypes, the cytokines they secrete, and the specific surface markers they present in (16, 17). M1​​​​​​-like phenotype is pro-inflammatory, suppressing cell proliferation and inducing inflammation, driving T helper 1 (Th1) type immune responses against different pathogens, such as bacteria, as well as leading inflammatory signaling pathways such as those activated by Toll-like receptors (TLRs) (18, 19). Conversely, M2-like phenotype is anti-inflammatory, linked with Th2-type responses found in conditions such as asthma and allergic reactions (20), facilitates cell proliferation and the repair of tissues ​​​​​​ (21). The classical M1 to M2 paradigm of macrophage polarization is basically their reaction to different cytokine surroundings: cytokines of Th1 origin such as interferon-gamma (IFN-γ) activate macrophages to M1 phenotype, while cytokines of Th2 origin such as Interleukin-4 (IL-4) and Interleukin-13 (IL-13) cause M2 polarization. Therefore, macrophage polarization not only contributes to shaping the type of immune response, but the resulting macrophage phenotype also reflects the local immune environment at the site of inflammation (22, 23).

#### 1.1.1 M1 macrophage phenotypes

**Figure 1:**
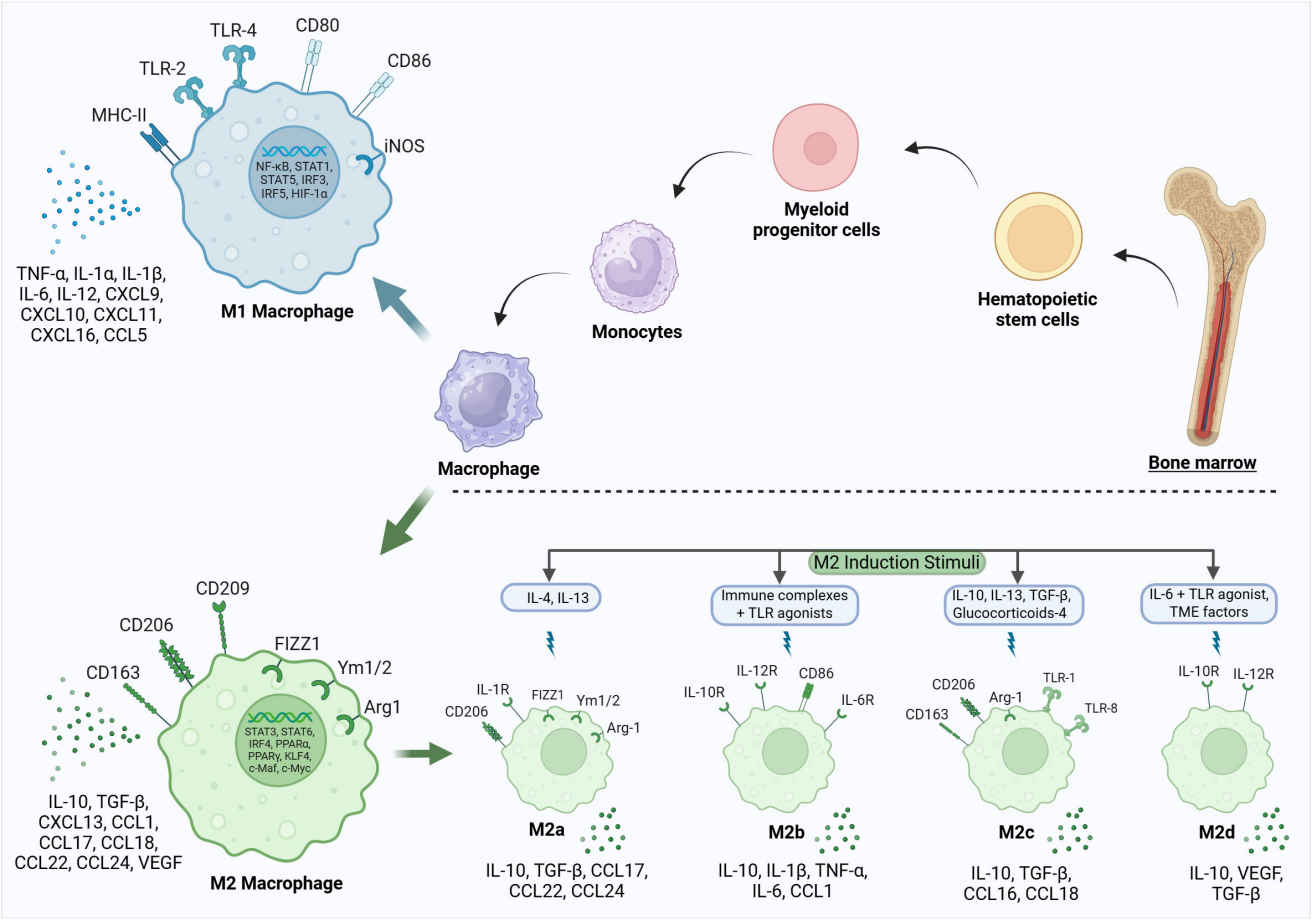
Macrophage biology and classification. Macrophage polarization into M1 and M2 phenotypes. M1 macrophages, classically activated, express surface markers including MHC-II, iNOS, TLR-2, TLR-4, CD80, and CD86, secrete pro-inflammatory cytokines (TNF-α, IL-1α, IL-1β, IL-6, IL-12, CXCL9, CXCL10, CXCL11, CXCL16, CCL5), and promote Th1 immune responses. Conversely, M2 macrophages, alternatively activated, express markers such as CD163, CD206, CD209, FIZZ1, and Ym1/2, secrete anti-inflammatory cytokines (IL-10, TGF-β, CCL1, CCL17, CCL24, VEGF), enhance phagocytosis, and support Th2 immune responses. Also, M2 macrophages can be divided into M2a, M2b, M2c, and M2d subtypes, each with unique stimuli, markers, and roles in tissue repair, anti-inflammation, and angiogenesis. Figures were created using BioRender (2026).

Pro-inflammatory (M1) macrophages are essential to initiate the immune response against pathogens. M1 macrophages are primarily activated by Th1 cytokines, particularly interferon-gamma (IFN-γ), as well as other stimuli including lipopolysaccharides (LPS), which is unique structural components of the outer membrane of gram-negative bacteria and potent activators of pro-inflammatory macrophage phenotype through Toll-like receptor 4 (TLR4) activation (24, 25). A core element of M1 macrophage polarization, is the surface marker expression including notably Cluster of Differentiation 80, 86 (CD80, CD86), Major Histocompatibility Complex class II (MHC-II) and Toll-like receptor 2 (TLR-2), TLR-4, and other key intracellular enzyme including inducible Nitric Oxide Synthase (iNOS) as show in **Figure 1** (26). They are also characterized by their production of various pro-inflammatory cytokines and chemokines. Common examples include Interleukin-6 (IL-6), Interleukin-12 (IL-12), Interleukin-1 alpha (IL-1α), Interleukin-1 beta (IL-1β), and Tumor Necrosis Factor alpha (TNF-α), all of which are essential in modulating the immune response. Promoting the production of inflammatory cytokines and coordinating host immunity in response to infection, these functions are important for both the initiation and maintenance of inflammation to effectively combat pathogens (27). These macrophages when activated produce also specific chemokines notably including CXCL9, CXCL10, CXCL11, CXCL16 and CCL5 that support Th1 inflammatory responses and also significantly enhance IFN-γ induced production of iNOS, and Reactive Oxygen Species (ROS) that results in the elimination of bacteria thereby providing antimicrobial effector molecules that can help lymphocytes in pathogen clearance (28). This set of pro-inflammatory factors augments the cytotoxicity and pathogen-fighting capacity of M1 macrophages. Also, excessive or prolonged M1 macrophage activity can lead to tissue destruction and chronic inflammation (16, 29). In addition, circulating monocytes can also be recruited to the site of inflammation, where they differentiate into different macrophages. These macrophages that infiltrate the area of inflammation are generally less mature, shorter-lived, and more responsive than residual populations (30).

#### 1.1.2 M2 macrophage phenotypes

M2 macrophages, which are generally considered anti-inflammatory, perform a variety of additional roles beyond simply suppressing inflammation. They can also be characterized by cell surface markers such as scavenger receptor, CD163, C-type lectin receptor, CD209 and mannose receptor, CD206 (31) as well as some intracellular proteins and enzyme including FIZZ1, Ym1/2, and arginase 1 (Arg1) (32, 33). The CD206 is observed highly due to its role to identify glycosylated molecules on apoptotic cells and components of the extracellular matrix, assisting the M2 macrophage in their ability to assist with tissue repair, debris removal, and regulation of the immune response without initially promoting a strong inflammatory response (34). This is prominent in M2 macrophage release of anti-inflammatory cytokines; notably, IL-10) and Transforming Growth Factor Beta (TGF-β), required to down regulate the inflammatory process and allow tissue repair. They also secrete IL-4, and IL-13 cytokines which promote Th2 responses that can mediate polarizing macrophages to an M2-like phenotype (35). M2 macrophages also secrete a family of chemokines, including but not limited to CCL17, CCL18, CCL22, and CCL24 to recruit the cells needed to facilitate tissue repair, as well as growth factors such as Vascular Endothelial Growth Factor (VEGF), and Platelet-Derived Growth Factor (PDGF) that are involved in angiogenesis, wound healing, and tissue remodeling (36).

Simultaneously, M2 macrophages also may attract immune cells by releasing chemokines such as CCL17 and CCL22 for the chemoattraction of Th2 cells (37). Besides that, they produce some matrix metalloproteinases (MMPs) including MMP-2 and MMP-9, which help in the breakdown of the already existing extracellular matrix (ECM) components, thus facilitating new ECM establishment and the migration of fibroblasts, endothelial cells, and immune cells that are the parts of tissue repair and regeneration (37). M2 macrophages can be categorized into M2a, M2b, M2c, and M2d; this reflects their transcriptional and functional diversity. Each M2 subset has a specific immunoregulatory and tissue homeostasis function in the immune microenvironment (38). IL-4 and IL-13 activate M2a macrophages, which are associated with tissue remodeling, wound healing, and regulating Th2 immunity. M2b macrophages are activated by immune complexes and TLR class agonists and provide both pro and anti-inflammatory activity when required. M2b macrophages can also participate in some levels of immunoregulation. M2c macrophages are induced by IL-10 or by TGF-β, or some glucocorticoids, and these cells have significant roles in mediating immunosuppression and assisting tissue repair. M2d macrophages are induced by IL-6 and by a TLR agonists are associated with angiogenesis and tumor progression (39). M2 macrophages also exhibit pathogenic potential; they can contribute to tumorigenesis and metastasis, creating challenges in advancing cancer research (40).

#### 1.1.3 Differences in macrophage common markers between humans and mice

Although the M1/M2 polarization framework has been extensively characterized in mouse models, many of the canonical markers used to define these states are not conserved across species (41). Direct extrapolation of murine macrophage phenotypes to human systems can be misleading, particularly in the context of translational and clinical research. One of the most notable discrepancies lies in the expression of enzymes involved in arginine metabolism. In mice, iNOS is a hallmark of pro-inflammatory (M1-like) macrophages, while Arg1 is strongly associated with anti-inflammatory (M2-like) macrophages. However, in human macrophages, iNOS expression is typically low or tightly regulated under comparable conditions, and Arg1 is not a reliable marker of alternative activation as shown in **Table 1** (42). Similarly, murine M2 markers such as Ym1/2 and FIZZ1 are absent in humans, further highlighting species-specific differences in macrophage characterization (37). In contrast, certain surface markers and cytokines are more consistently conserved between species, although their expression patterns may still vary depending on context. For instance, markers such as CD206 and CD163 are commonly used to identify anti-inflammatory or tissue-associated macrophages in humans, whereas their expression in mice may differ in magnitude or functional implication (45). Pro-inflammatory markers including CD80, CD86, and MHC-II (HLA-DR in humans) molecules are present in both species, but their regulation and co-expression profiles are influenced by tissue environment and signaling conditions (43, 44). Importantly, advances in transcriptomic and single-cell analyses have demonstrated that macrophage activation states are highly heterogeneous and shaped by complex microenvironmental signals in both humans and mice. These studies reveal that even when similar markers are expressed, the underlying gene regulatory networks and functional outputs may differ between species. Consequently, reliance on a limited set of canonical markers may oversimplify macrophage biology and obscure critical interspecies of differences.

**Table 1:** Differences in macrophage common marker expression between humans and mice.

Subtypes	Common markers	Expression in mouse	Expression in human	Ref.
M1 subtype	iNOS	Strong, canonical M1 marker	Low/variable, tightly regulated	(42)
	CD80	Expressed	Expressed	(43, 44)
	CD86	Expressed	Expressed	(43, 44)
	MHC-II (I-A/I-E)	Strong expression	HLA-DR, HLA-DP, HLA-DQ	(43, 44)
M2 subtype	Arginase-1 (Arg1)	Strong M2 marker	Low/variable, not reliable	(37)
	Ym1/2	Expressed	Absent	(37)
	FIZZ1	Expressed	Absent	(45)
	CD206	Expressed	Expressed	(45)
	CD163	Expressed (variable)	Strong marker	(46, 47)

### 1.2 Signaling pathways and biomarkers associated with M1 and M2 polarization

**Figure 2:**
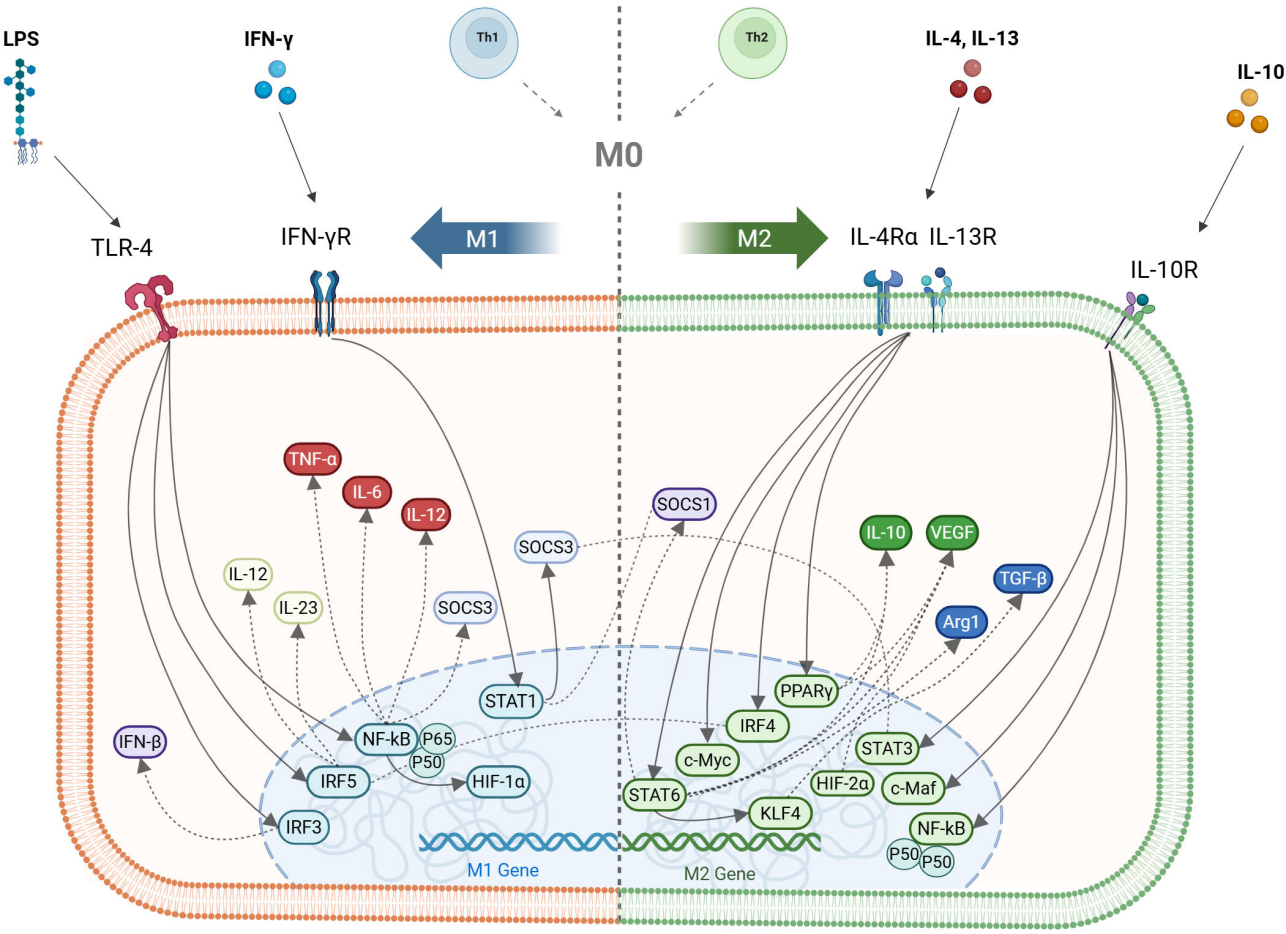
Signaling pathways regulating macrophage polarization. M1 or M2 phenotypes are governed by distinct signaling pathways. M1 polarization: LPS activates TLR4, triggering NF-κB and MAPK (ERK, JNK, p38) pathways to induce pro-inflammatory cytokines (TNF-α, IL-6, IL-12), while IFN-γ signals through JAK-STAT1 and transcription factors IRF3/IRF5 enhance inflammatory gene expression. M2 polarization: IL-4 and IL-13 activate JAK-STAT6 and KLF4, promoting anti-inflammatory genes (Arg1 e.g.), and IL-10 signals via JAK1-STAT3 to suppress inflammation. Additional transcription factors IRF4, c-MYC, c-Maf, and HIF-2α, together with PPARγ and TGF-β signaling, drive tissue repair, metabolic adaptation, and angiogenesis (VEGF production). NF-κB, nuclear factor kappa B; STAT, signal transducer and activator of transcription; IRF, interferon regulatory factor; KLF4, Kruppel-like factor 4; c-MYC, MYC proto-oncogene; c-Maf, transcription factor Maf; HIF-2α, hypoxia-inducible factor 2 alpha; PPARγ, peroxisome proliferator–activated receptor gamma. Figures were created using BioRender (2026).

The polarization of macrophages into either M1 or M2 phenotype is regulated through multiple complexes signaling pathways that form the networks of molecular interactions which are fundamental in driving macrophage polarization (48). These signaling pathways, activated in response to various external stimuli such as cytokines, growth factors, or microbial components, are associated with the initiation of transcription of gene clusters and their activity in a range of physiological and pathological contexts (49). For instance, M1 macrophage polarization involves activation of the NF-κB signaling pathway, leading to the upregulation of pro-inflammatory cytokines such as TNF-α, IL-6, and IL-12. Stimulation with IFN-γ activates the JAK-STAT1 signaling pathway to stimulate expression of M1 genes, as depicted in **Figure 2** (50, 51). The NF-κB pathway is a master regulator of M1 activation and coordinator of the production of cytokines and chemokines and other enzymes mediating inflammation. Additionally, transcription factors such as IRF3 and IRF5 are also contributed to the expression of IL-12 and IL-23 and other pro-inflammatory genes (52). In addition, the MAPK pathway (including the major kinases ERK, JNK and p38 MAPK) can be activated by inflammatory signals downstream of the TLRs and induce the transcription of pro-inflammatory genes behind M1 macrophage response. Thus, these signaling pathways both lead to an M1 phenotype macrophage and elevate inflammation. Likewise, the NLRP3 inflammasome can be activated by PAMPs or Damage-Associated Molecular Patterns (DAMPs) to cleave caspase-1 and consequently mature and release IL-1β and IL-18 to drive inflammation and enhance the antimicrobial response (53).

Conversely, the transition to the M2 macrophage phenotype is facilitated through a distinct array of signaling pathways. The JAK-STAT pathway is indispensable for M2 polarization and is stimulated by IL-4 and IL-13, primarily through the action of STAT6 and KLF4 (54, 55). This signaling cascade occurs due to IL-4 and IL-13 interacting with their specific receptors which initiates the transcription of genes associated with tissue repair, anti-inflammatory function, and upregulation of distinctive markers such as Arg1 as shown in **Figure 2**. IL-10 is a primary promoter of M2 polarization, as once this cytokine binds to its receptor, JAK1 is activated, followed by STAT3 activation that enhances anti-inflammatory cytokine production and suppresses pro-inflammatory responses. There are additional transcription factors that regulate M2 polarization such as, c-MYC, IRF4, NF-κB p50 homodimers, c-Maf, and HIF-2α which aid in regulating gene programs that drive anti-inflammatory responses, tissue repair, and metabolic adaptation to hypoxic conditions (52, 56). By activating PPARγ, a nuclear receptor activated by certain metabolites, macrophage metabolism is channeled into anti-inflammatory metabolism; TGF-β pathway signaling promotes wound healing and fibrosis mediated by altered gene expression of the cell growth process and ECM production. M2 macrophages induce angiogenesis via the production of growth factors such as VEGF to facilitate tissue repair and remodeling (57).

### 1.3 Heterogeneity, plasticity, and repolarization dynamics of macrophage phenotypes

Macrophages are highly dynamic cells characterized by pronounced phenotypic plasticity, enabling them to rapidly adapt to diverse microenvironmental cues. This plasticity reflects their ability to modify gene expression programs, signaling pathways, and effector functions in response to stimuli such as pathogens, cytokines, and tissue-derived factors (7, 58). Their functional phenotypes are therefore shaped by complex and overlapping environmental signals, resulting in gradual, reversible, and context-dependent transitions (59). Macrophage heterogeneity is further influenced by their developmental origin and tissue-specific microenvironment (60). Tissue-resident macrophages can arise from embryonic progenitors established during early development, whereas inflammatory macrophages are frequently derived from circulating monocytes recruited from the bone marrow (61). These distinct ontogenies contribute to differences in transcriptional programs, metabolic profiles, self-renewal capacity, and responsiveness to external stimuli. In addition, local tissue-derived factors continuously shape macrophage identity, resulting in specialized populations adapted to the physiological requirements of specific organs such as the liver, lung, brain, skin, and tumor microenvironment (62). Consequently, macrophages isolated from different anatomical sites or derived through distinct experimental systems, including peripheral blood monocyte-derived macrophages and tumor-associated macrophages, may exhibit differential responses to identical stimuli due to intrinsic developmental programming and microenvironmental conditioning (60). M0, M1, and M2 classification system is a simplified framework, for discussions in experimental and *in vitro* settings (63). Instead, macrophages exhibit a range of intermediate and mixed phenotypes that reflect their ability to integrate multiple signaling inputs simultaneously, particularly *in vivo* conditions (64). These transitions include bidirectional shifts between M1-to-M2 or M2-to-M1 states, as well as reversion to the resting M0 state, and are collectively referred to as macrophage repolarization (65, 66). This is an important process, explaining how the immune system adapts and functions in response to different stimuli. For example, the shifting of M1 into an M2 phenotype as part of an infection or tissue injury indicative of their functional role to progression of inflammatory conditions or stimulate regulatory processes to restore homeostatic balance (67). The impact of uncontrolled inflammation or inappropriate dampening of inflammation thus indicates that both M1 and M2 macrophages have the propensity to promote homeostasis in the immune response in pathological processes. Moreover, increasing evidence demonstrates that macrophages frequently co-express markers traditionally associated with both M1 and M2 phenotypes at the same time. Macrophages exposed to combinations of pro-inflammatory signals such as IFN-γ or TNF-α, alongside anti-inflammatory signals such as IL-4 or IL-13 can adopt a hybrid phenotype which functionally represents both M1 and M2 states. Single-cell RNA sequencing (scRNA-seq) of mouse Bone Marrow-Derived Macrophages (BMDMs) treated with LPS + IFN-γ, IL-4, or their combination revealed that genes activated by LPS + IFN-γ are also co-expressed with IL-4-induced genes, demonstrating simultaneous activation of opposing programs within individual cells (68). Similarly, other co-stimulation studies have reported the co-expression of markers associated with both inflammatory and regulatory macrophage phenotypes in disease-associated macrophages, further supporting the concept that macrophage activation exists along a functional spectrum rather than within strictly separated categories (69, 70).

## 2 Macrophage polarization in disease contexts

### 2.1 Infectious diseases

During infectious diseases, macrophage polarization is a key determinant of host defense, directing the immune response toward pathogen clearance while influencing the degree of inflammation and tissue damage (67). For instance, some studies showed that macrophage polarization from the M0 to the M1 phenotype was induced by stimulation of BMDMs with outer membrane vesicles derived from *Fusobacterium nucleatum*, which contain bacterial components such as LPS outer membrane proteins that act as PAMPs to activate TLR mediated signaling pathways. This activation triggered pro-inflammatory signaling cascades, including NF-κB activation, resulting in the upregulation of M1-associated markers such as IL-1β, IL-6, TNF-α, and iNOS (71). Such pro-inflammatory M1 phenotype, contributing to increased inflammation, alveolar bone loss, and periodontal tissue destruction in the *F. nucleatum* induced periodontitis model (72). As shown in **Table 2**, cytokines and effector molecules from chronically overactivated M1 macrophages can cause collateral tissue injury and contribute to immunopathology, which must be resolved to prevent chronic inflammation.

**Table 2:** Main role of Macrophages in infectious diseases.

Macrophages	Key induction signals	Key cytokines	Main role in infectious disease (73)
M0 subtype	Baseline state, no strong polarizing signal	Low basal cytokines	Precursor macrophages survey tissue and polarize into M1 or M2 depending on the pathogen encountered.
M1 subtype	IFN-γ, LPS, TNF-α	IL-1β, IL-6, IL-12, TNF-α, ROS, NO	Eliminate pathogens via pro-inflammatory responses; promote Th1 responses; but excess can cause tissue damage.
M2 subtype	IL-4, IL-13, IL-10, glucocorticoids	IL-10, TGF-β, Arg1, VEGF	Resolve inflammation and repair tissue; promote Th2 responses; some pathogens also exploit M2.

At this stage, macrophages must transition to an M2-like state to help resolve the infection and promote tissue healing. Activation of the M2-like state promotes the suppression of inflammation through the production of anti-inflammatory cytokines, particularly IL-10 and TGF-β (74). Hence, ​​​​​ polarization of macrophages determines not only the infectious diseases’ progression but also the eventual healing. Therefore, macrophages’ plasticity and the possibility to switch between M1 and M2 states allow the immune system to not only eliminate the pathogens but also to normalize the tissue which highlights the importance of macrophage polarization in the infectious diseases’ severity and its progression.

### 2.2 Chronic inflammatory and autoimmune diseases

**Figure 3:**
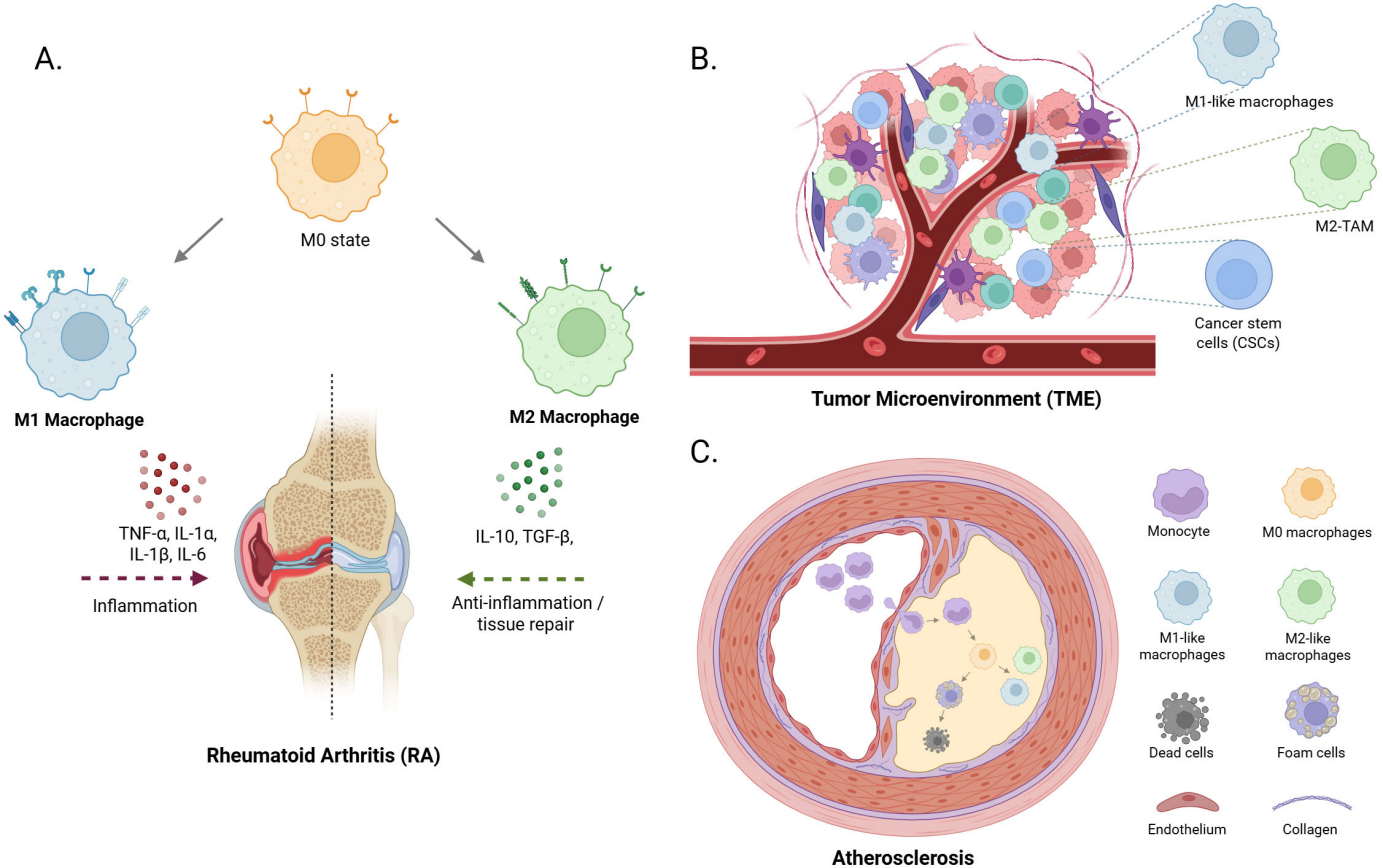
Macrophage polarization in disease contexts. **(A)** In rheumatoid arthritis (RA), persistent M1 macrophages sustain inflammation and tissue damage. **(B)** In cancer, M2-like tumor-associated macrophages (TAMs) promote immunosuppression, angiogenesis, and tumor progression. **(C)** Macrophage polarization in Atherosclerosis. M1 macrophages drive inflammation and tissue damage, while M2 macrophages promote repair and resolution; imbalance affects disease progression. Figures were created using BioRender (2026).

Many chronic inflammatory diseases, including autoimmune disorders such as experimental autoimmune encephalomyelitis (EAE) and rheumatoid arthritis (RA), are characterized by persistent M1 polarization. In these conditions, macrophages, particularly M1 subtypes, continuously secrete pro-inflammatory cytokines, sustaining local inflammation, tissue damage, and the recruitment of autoreactive immune cells as depicted in **Figure 3A**. Systemically, this pro-inflammatory environment can increase disease severity and contribute to immune dysregulation (75). For example, microglial polarization toward the pro-inflammatory M1 phenotype plays a crucial role in the pathogenesis of EAE by promoting neuroinflammation and demyelination, while the anti-inflammatory M2 phenotype is underrepresented (76). Also, Astragalus polysaccharides (APS) can restore microglia balance by inhibiting M1 polarization and promoting M2 polarization, thereby alleviating disease severity (77). These findings highlight that targeting microglia polarization may represent a promising therapeutic strategy in neuroinflammatory diseases. Another study found that macrophage polarization plays a key role in the pathogenesis of (RA), as pro-inflammatory M1 macrophages in inflamed joints secrete cytokines that sustain local inflammation, drive tissue damage, and recruit autoreactive immune cells, thereby exacerbating disease severity (76). They showed folic acid-modified silver nanoparticles (FA-AgNPs) to selectively induce M1 macrophage apoptosis and promote M2 polarization, effectively reducing joint inflammation and tissue damage in RA models. These findings underscore macrophage polarization as a potential target for RA therapy (78). On the other hand, M2 macrophages are generally associated with resolving inflammation and promoting tissue repair in RA (19). They secrete anti-inflammatory cytokines and growth factors, such as IL-10 and TGF-β, which help limit immune-mediated tissue damage and support joint regeneration.

### 2.3 Cancer and tumor microenvironment

Macrophage polarization within the tumor microenvironment is a critical determinant of tumor progression, immune evasion, and therapeutic response. Classically−activated M1 macrophages exhibit pro−inflammatory and tumor−suppressive functions, producing cytokines that activate cytotoxic immune cells and inhibit tumor growth. In contrast, alternatively−activated M2 macrophages, which dominate as tumor−associated macrophages (TAMs) as illustrated in **Figure 3B** in TME, support tumor growth by secreting immunosuppressive factors and promoting angiogenesis, remodeling the extracellular matrix and suppressing antitumor immunity (79, 80). In addition, cancer stem cells (CSCs) also actively regulate macrophage polarization within the TME, contributing to the maintenance of an immunosuppressive, tumor-promoting niche (81). The transformation of this phenotype is a result of signals coming from the tumor such as IL-4, IL-10, and TGF-β, which cause the macrophages to depart from their usual state (82). Also, tumor-associated macrophages not only show increased expression of the surface markers CD206, CD163, and Arg1 but also, compared to macrophages from healthy tissues, they produce constant anti-inflammatory cytokines and, in addition, have an upregulated secretion of VEGF and MMPs. These molecules, in combination, lead to tumor growth, angiogenesis, and extracellular matrix remodeling (83, 84). Studies found that reprogramming tumor-associated macrophages (TAMs) from the tumor-promoting M2 phenotype to the tumor-suppressing M1 phenotype can enhance antitumor immunity. Mannose- functionalized, macrophage-membrane-coated upconverting nanoparticles (UCNPs) were designed to selectively target M2-type TAMs in hypoxic breast tumors. Upon internalization, the nanoparticles generated ROS and released oxygen, promoting M1 polarization of TAMs (85). These reprogrammed M1 macrophages secreted pro-inflammatory cytokines, reshaping TME, and suppressing tumor growth both *in vitro* and *in vivo*. These findings highlight that TAM polarization represents a promising strategy for cancer immunotherapy by converting immunosuppressive tumor niches into pro-inflammatory, tumor-suppressive environments.

### 2.4 Tissue repair, fibrosis and atherosclerosis

Macrophage polarization plays a significant role in tissue repair and progression of chronic diseases including fibrosis and atherosclerosis. Circulating monocytes are recruited to sites of tissue injury or inflammation, where they infiltrate the affected tissue and differentiate into macrophages in response to local microenvironmental signals, as illustrated in **Figure 3C**. For instance, during the recovery phase of tissue injury, macrophages undergo a phenotypic transition from M1 to M2 phenotype. M2 macrophages secrete a variety of growth factors, including VEGF and TGF-β, which promote angiogenesis, extracellular matrix remodeling, and tissue repair (86). These are very important functions as they renew tissue integrity and balance after injury or inflammation (87). On the other hand, macrophage polarization determines the fate of plaque in atherosclerosis, affecting plaque formation and stability. In early atherosclerotic lesions, M1 macrophages prevail, which causes a series of events by releasing pro-inflammatory cytokines, ROS, and matrix-degrading enzymes which lead to endothelial dysfunction and plaque instability (88). In contrast, M2 macrophages support the consolidation of plaque by promoting efferocytosis and thus the elimination of apoptotic cells, as well as the production of anti-inflammatory cytokines which are inflammation suppressive. Furthermore, the imbalance in these states can lead to pathological remodeling, contributing to fibrosis in organs such as the liver and heart, and also plaque formation in arteries during atherosclerosis (89). Other studies have shown that macrophage polarization plays a central role in hepatic fibrosis, acting as a key link between inflammation and ECM deposition (90). Kupffer cells can adopt both M1 or M2 phenotypes, with M1 macrophages promoting liver injury and M2 macrophages enhancing fibrosis through ECM accumulation. Also, Chitooligosaccharides (COS) alleviated hepatic fibrosis by modulating Kupffer cell polarization, reducing the excessive activation of both M1 and M2 macrophages, and limiting the activation of hepatic stellate cells (90). These studies reinforced the importance of macrophage polarization in liver fibrosis and suggest that targeting the balance between pro-inflammatory and anti-inflammatory macrophage phenotypes represents a promising strategy to restore immune homeostasis and mitigate fibrotic progression.

### 2.5 Translational implications of macrophage plasticity in disease and therapy

Targeting macrophage functional states represents a promising therapeutic strategy across diverse pathological conditions; however, its translational success depends on carefully accounting for the context-dependent and spectrum-based nature of macrophage activation (91, 92). For example, as explained in Section 2.3, the functional heterogeneity of TAMs has spurred therapeutic strategies for cancer, including targeting macrophage recruitment and polarization, blocking myeloid inhibitory signals, i.e., through myeloid checkpoint inhibition, and engineering macrophages for cell-based immunotherapy (93). The intrinsic plasticity provides significant therapeutic opportunities but also introduces major challenges for clinical translation, particularly in complex and evolving disease settings. A central limitation in macrophage-targeted therapy is the marked variability of macrophage responses across disease stages, tissue environments, and cellular ontogeny (60). Consequently, identical interventions may produce divergent or even opposing outcomes depending on the inflammatory milieu, timing of administration, and local tissue signaling context. Also, early-stage inflammatory environments may favor pro-inflammatory activation, whereas later stages may promote reparative or immunoregulatory responses (94). In addition, macrophage heterogeneity directly shapes disease outcomes beyond cancer as well. In tuberculosis, the divergent responses of alveolar and interstitial macrophage lineages within the granuloma are governed by ontogeny and epigenetic programming rather than a simple M1/M2 activation state, with direct implications for optimizing both vaccines and drug regimens (95). The inherent plasticity, while therapeutically attractive, remain a concern in clinical translation: macrophage-based cell therapies, including *ex vivo*-polarized and CAR-macrophage approaches, have entered clinical trials across conditions including kidney disease, stroke, arterial disease, and cancer, with outcomes demonstrating both promise and context-dependent variability (5). In joint diseases such as rheumatoid arthritis and osteoarthritis, macrophage reprogramming strategies have demonstrated immunomodulatory and anti-inflammatory effects in both preclinical studies, however, clinical translation remains incomplete, with dedicated clinical trials still needed to validate macrophage-targeted approaches in these condition (96). Additionally, other experimental evidence from inflammatory disease models indicates that macrophage-targeted interventions can simultaneously suppress pro-inflammatory signaling while modulating tissue remodeling and repair pathways, demonstrating that phenotypic switching alone does not reliably predict functional or clinical outcomes (97, 98). Collectively, these studies emphasize that macrophage-targeted therapies must be interpreted within a framework of functional heterogeneity rather than binary polarization states. Moreover, systemic or non-specific targeting approaches may lead to off-target immune effects due to the widespread distribution, plasticity, and functional diversity of macrophage populations across tissues (97). Despite these limitations, several therapeutic approaches continue to demonstrate translational promise, including cytokine modulation, targeted inhibition of key inflammatory signaling pathways, metabolic reprogramming strategies, and emerging nanotechnology-based delivery systems designed for macrophage-specific targeting and functional reprogramming (99, 100). Importantly, clinical and translational evidence suggests that successful macrophage-targeted therapies depend not on rigid M1/M2 switching, but on precise spatiotemporal control of treatment, disease stage awareness, and modulation of the local immune microenvironment, reflecting a shift from phenotype-based targeting toward function- and context-driven therapeutic approaches.

## 3 Current methods for inducing macrophage polarization study

### 3.1 Types of macrophages cell lines for *in vitro* polarization research

Macrophage research heavily relies on *in vitro* methods to uncover the diverse functions and behaviors of these cells. These methods allow precise adjustment of cellular growth conditions to direct macrophages toward either M1 or M2 phenotypes, which exhibit distinct functional properties and participate differently in immune responses. A critical consideration is the choice of cell models, whether a benchmark cell line or a primary cell line, as different models can reveal unique aspects of macrophage function. As shown in **Table 3**, numerous cell lines are available for macrophage polarization studies. Human cell lines are frequently used to investigate human-specific immune responses. For instance, human monocyte-derived macrophages (hMDMs), isolated from blood monocytes, provide a relevant model for human-focused research (109). THP-1 cells, derived from the human monocytic lineage, can differentiate into macrophage-like cells upon exposure to specific inducing agents, making them attractive for experimental studies (74). Additionally, macrophages derived from human induced pluripotent stem cells (iPSCs) (110) are increasingly employed for polarization studies. Since many *in vivo* experiments are conducted in mice, initial *in vitro* studies are often performed with mouse cell lines to elucidate underlying mechanisms. Bone marrow-derived macrophages (BMDMs) obtained from mice are particularly valuable due to their relevance for *in vivo* simulations (111). Other widely used mouse cell lines include RAW 264.7, J774, and also immortalized BMDMs, which provide stable and reproducible experimental outcomes. The murine RAW 264.7 cell line is especially popular because of its adaptability to diverse experimental conditions (112). J774 cells are similarly recognized for their reliability and reproducibility (113). Immortalized BMDMs offer a close-to-primary model with enhanced genetic stability (114), while peritoneal macrophages (115) provide another mouse-derived option for polarization studies. Hence, selection of a particular cell type depends on factors such as the potential for genetic manipulation, experimental scalability, and relevance to either human or mouse biology.

**Table 3:** Commonly used macrophage cells for polarization study.

#	Macrophage cell lines	Source and definition	Ref.
1	Bone Marrow-Derived Macrophages (BMDMs)	Primary cells are isolated from the bone marrow of murine. BMDMs are commonly used in studies for their similarity to natural conditions.	(101)
2	Immortalized Bone Marrow-Derived Macrophage (iBMDM)	Genetically modified and mouse bone marrow–derived, these cells have extended lifespans compared to traditional BMDMs, making them ideal for experiments that require stable, long-term macrophage cultures.	(102)
3	Human Monocyte-Derived Macrophages (hMDMs)	Sourced from human blood monocytes, hMDMs are essential for studying human-specific immune responses in macrophage biology.	(103)
4	THP-1	Human monocytic cell lines that can differentiate into macrophage-like cells using certain substances including PMA (phorbol 12-myristate 13-acetate).	(104)
5	J774	Mouse macrophage cell lines which are used in a wide range of immunology and pathology research. Their easy maintenance makes them suitable for investigating macrophage activities to different stimuli.	(105)
6	RAW 264.7	The leukemic monocyte/macrophage cell line is derived from mice. Culturing these cells is manageable, making them a popular option. However, they may not entirely reflect the characteristics of primary cells.	(106)
7	Macrophages derived from human induced pluripotent stem cells (iPSCs)	These cells obtained from human-induced pluripotent stem cells (iPSCs) are increasingly used. They offer the potential to study patient-specific responses and are important for personalized medicine research.	(107)
8	Peritoneal Macrophages	Derived from the peritoneal cavity, these cells are used in studies which are related to abdominal infections, inflammation, and gut-related immune responses.	(108)

### 3.2 Co-culture systems

Macrophage polarization studies using co-culture systems are specialized techniques designed to replicate interactions between macrophages and other cell types. Polarization states in co-culture systems are complex and diverse, driven primarily by cytokines and signaling molecules exchanged with adjacent cells. Macrophages can be co-cultured with various cell types, including tumor cells, fibroblasts, endothelial cells, and other immune cells such as T cells or dendritic cells (which may also be cultured separately). This approach effectively simulates the complex cellular microenvironment found in tissues during physiological processes, such as wound healing, or pathological conditions, such as inflammation and tumor progression, thereby enhancing the relevance and applicability of experimental outcomes to *in vivo* biology (116). As explained previously, cytokines such as IFN-γ and TNF-α drive macrophage polarization toward the pro-inflammatory M1 phenotype, whereas IL-4, IL-10, and IL-13 induce polarization toward the anti-inflammatory M2 phenotype under this co-culture conditions, thereby mimicking key aspects of *in vivo* macrophage biology. These interactions involved direct cellular contact or the secretion of cytokines, chemokines, growth factors, and other signaling molecules (117). For instance, in a co-culture setting of macrophages and tumor cells, M1 macrophages secrete inflammatory cytokines that inhibit tumor growth, whereas M2 macrophages may promote tumor progression and suppress immune responses. In addition, tumor cells may secrete particular factors that lead to the conversion of macrophages to a tumor-promoting M2 phenotype, as a result, a phenomenon that is regularly observed in the TME (118). Such co-culture systems are of essential methods to researchers who are eager to find out the extent to which macrophages regulate other cells’ behavior, a factor that is instrumental in cancer research for unraveling the intricate interactions within the TME involving cancer cells, immune cells, and stromal cells. Investigating these dynamics is central to unveiling tumor progression, metastasis, and resistance to treatment. Co-culture systems also play a significant role in research on wound healing, fibrosis, and various inflammatory and autoimmune diseases, greatly facilitating the development of more effective and simple therapeutic strategies that target macrophage-mediated responses in different pathological conditions.

### 3.3 Isolation of primary macrophages

**Figure 4:**
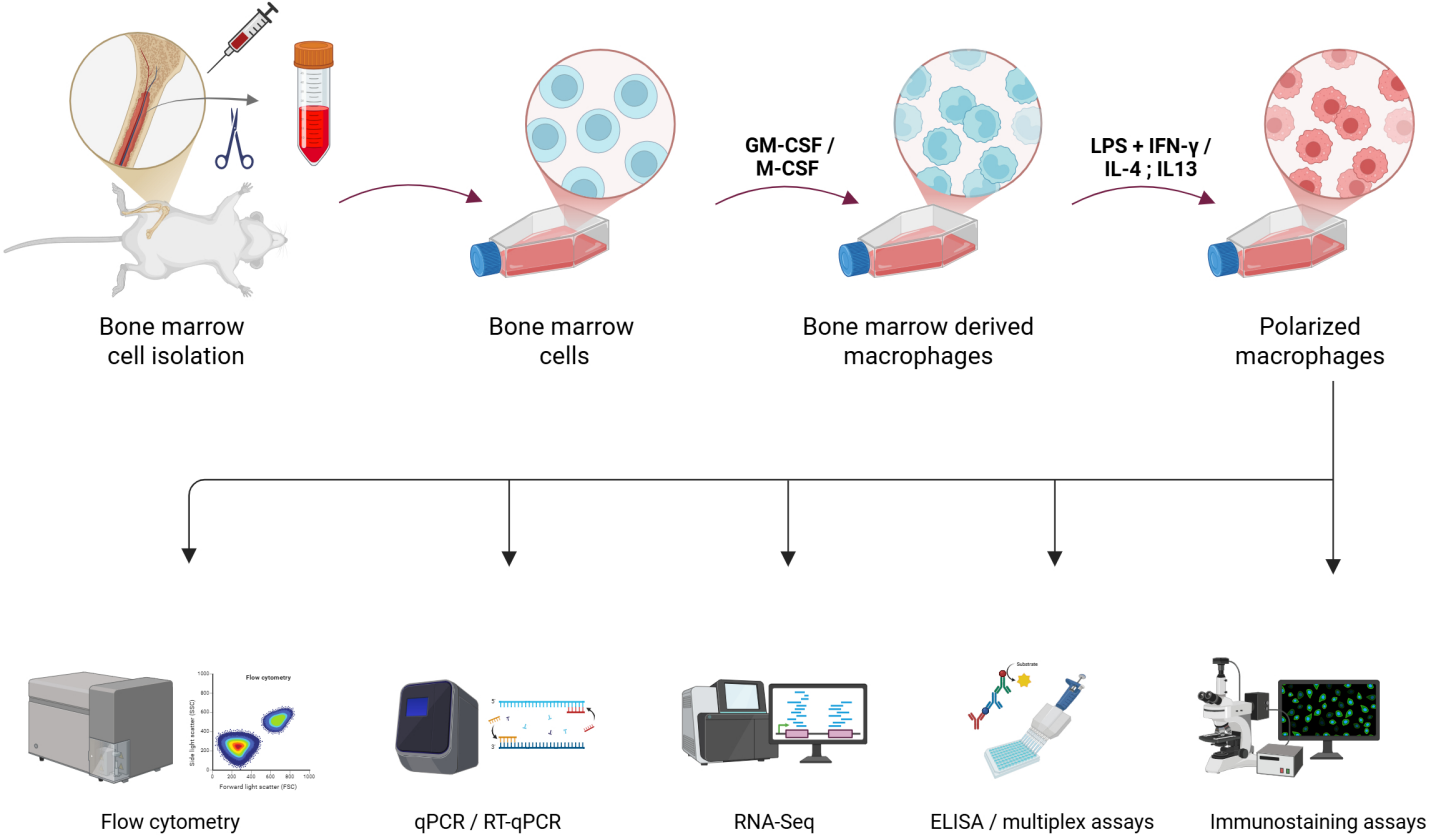
Schematic of primary macrophage (BMDM) isolation, differentiation, and stimulation. Bone marrow cells are harvested from murine femurs and tibiae under sterile conditions. Progenitor cells are differentiated with GM-CSF to generate M1-like macrophages or M-CSF to generate M2-like macrophages. Differentiated BMDMs initially reside in a baseline M0 state and can be further polarized into M1 (LPS + IFN-γ) or M2 (IL-4 + IL-13) phenotypes. Cell surface markers and functional characteristics can be assessed using flow cytometry, qPCR, ELISA/multiplex assays, RNA-Seq and immunofluorescence imaging, e.g. Figures were created using BioRender (2026).

The isolation of primary macrophages from mice is an important process in immunological studies and provides cells for *in vitro* experiments. The process involves harvesting cells, such as monocytes or hematopoietic stem cells, from bone marrow or peripheral blood. Then, those stem progenitor cells are incubated with the addition of differentiation factors, such as Macrophage Colony-Stimulating Factor (M-CSF) or Granulocyte-Macrophage Colony-Stimulating Factor (GM-CSF) to guide their differentiation into macrophages as illustrated in **Figure 4**. The type of macrophages that will be generated depends on the growth factor that is used for the cells. M-CSF mainly results in the differentiation of macrophages that exhibit the characteristics of tissue-resident or anti-inflammatory M2-like macrophages. GM-CSF, on the other hand, imparts the macrophages with pro-inflammatory M1-like properties. Scientists can direct the polarization of progenitor cells by employing these cytokines and in this way, they can investigate the roles of macrophages under physiological and pathological conditions, infection, inflammation, autoimmune disorders, and cancer etc. Once differentiation is done, macrophages are usually assumed to be in a resting M0 state from which they can be additionally polarized to M1 or M2 phenotypes by the influence of further extra stimuli, notably such as LPS and IFN-γ for M1 or IL-4 and IL-13 for M2 states. After that, phenotypic characterization and functional assays are performed to identify the polarization state and to evaluate the implementation of the activities, such as cytokine production, phagocytosis, antigen presentation, by the main laboratory methods such as flow cytometry, qPCR, cytokine profiling, or immunostaining. In addition, macrophage culture methods *in vitro* offer a better controlled condition for the study of macrophage biology, but it is also necessary to be aware that these cannot entirely mimic the complicated *in vivo* condition where macrophages are continuously subjected to a mixture of tissue-specific signals, extracellular matrix components, and cellular interactions. But these strategies remain essential for understanding the mechanisms of macrophage development, polarization, and functional specialization, and they serve as a basis for translational studies aimed at regulating macrophages for therapeutic use.

### 3.4 *In vivo* analysis methods

*In vitro* macrophage polarization enables the study of diverse macrophage activities under controlled conditions; however, it does not fully capture the complexity of polarization *in vivo*, where multiple interactions and stimuli influence macrophage behavior. Hence, many results obtained from *in vitro* experiments are complemented or expanded with *in vivo* studies to get a more thorough conception of macrophage biology, including their variable reactions to tissue-specific and systemic factors (119). The research on macrophage polarization under normal and disease conditions is mainly carried out using murine *in vivo* models of inflammation and diseases, that is, mice or rats (120, 121). Common methods for assessing, including intravital microscopy and fluorescence or bioluminescence imaging (122), as well as PET/CT or MRI combined with advanced tracers (123), enable accurate localization and quantification of macrophage behavior and polarization changes *in vivo* condition. Immunohistochemistry or immunofluorescent approaches enable depiction of differently polarized macrophages’ localization within tissues (*see Section 5*) (124). Also, tissues can be collected for broader morphological analyses, facilitating the evaluation of membrane surface markers and intracellular cytokines to identify macrophage polarization states. Furthermore, to identify the genes that play a role *in vivo* macrophage polarization, scientists frequently resort to genetically modified mouse strains that have specific gene knockouts (KO). These specialized KO mouse models are tailored to interfere with certain gene regulations that govern different macrophage polarization states (M1 or M2) and are being utilized in numerous scenarios such as the examination of immune responses, inflammation, tissue repair, and disease mechanisms (125). For instance, Stat6 KO mice are devoid of the transcription factor STAT6 which is indispensable for IL-4/IL-13-mediated M2 macrophage polarization and hence represent an excellent tool to investigate the mechanism of defective alternative macrophage activation (126). Such models serve as a significant source for studying changes in macrophage behavior and function after performing genetic modification experiments and thus shedding light on their contribution in a variety of normal and disease conditions. Altogether, these *in vivo* strategies broaden the *in vitro* studies horizon and collectively present a multi-faceted picture of macrophage biology revealing their functional plasticity depending on the context of health or illness.

## 4 Challenges and recommended protocols for experimental consistency

### 4.1 Considerations for macrophage cell type selection, confluency and handling

In general, immortalized cell lines are suitable for high throughput studies since they are readily culturable, reproducible, and consistent; nevertheless, they may not reflect the functional aspect of primary macrophages (127). On the other hand, primary macrophages more closely mimic *in vivo* biology and exhibit tissue-specific, physiologically relevant responses, providing deeper insight into macrophage behavior. However, they require a labor-intensive isolation process, have a limited lifespan under culture conditions, and may be affected by variability (128). Hence, choosing macrophage sources, careful recording of the isolation and culture methods, as well as baseline characterization are the necessary steps in reproducibility achievement and making the substantial comparison possible between various research works ​​​​​​ (129). Also, the cell density during differentiation, including the cellular confluency at the time of induction, is of utmost importance as it can significantly alter the phenotype of macrophage such as bone marrow-derived macrophages (130). Cells grown too densely may suffer from limited nutrient and cytokine diffusion, reduced receptor accessibility, and altered paracrine signaling, while very sparse cells may lack sufficient cell–cell interactions necessary for optimal functional responses (130). A way to do this properly is to prepare macrophages at the best confluency for immortalized lines or at the end of differentiation of primary macrophages, thereby ensuring that all cells are equally exposed to the inducers without causing stress or overcrowding. They can also adjust cytokine concentrations depending on cell density in order to keep signaling at an effective level and produce reproducible outcomes (131). Moreover, *in vitro* experiments with macrophages require the use of different detachment methods depending on the downstream applications and the necessity to keep surface markers intact. Scraping is a gentle, mechanical method that not only detaches cells but also does not significantly change surface proteins, thus making it better for specific marker analysis by flow cytometry or immunostaining. On the other hand, trypsin or some other enzymatic detachment methods are very efficient in detaching most cells but at the same time can drastically reduce marker integrity if exposure is prolonged, hence this method is only appropriate for experiments where the surface protein integrity is not needed, such as RNA or protein extraction or functional studies. Taking the right detachment step will, on the one hand, guarantee maximum cell recovery, and on the other, it will make results reliable in subsequent analyses ​​​​​​ (132, 133).

### 4.2 Animal model influences: strain and origin

The​​​​​​ species origin of the cells is important factor to consider in macrophage polarization studies. This is because the biomarkers, cytokine profiles, and functional attributes related to polarization differ markedly between species. For instance, macrophages derived from mice and humans display distinct cellular characteristics (134). Typically, human macrophages can be derived from the monocytes found in the blood, while the mouse macrophages originate either from the progenitors in the bone marrow or the tissue-resident populations, such as peritoneal macrophages. Such variations are reflected in the differential production of cytokines, expression of surface markers, and stimulability, as well as in the dissimilarities of metabolic activity and cellular plasticity (135). Knowledge of these distinctions is necessary for the correct reading of experimental data and for the transfer of insights from the mouse models to human biology​​​​​. Moreover, the variation has instigated discussion within the scientific community with many papers emphasizing either the fundamental similarities or the differences of macrophages between humans and mice (14). For instance, strain-specific differences in murine macrophages could deeply impact the cellular functions, and thus it is worth pointing out that one should be very careful in planning experiments. This can be exemplified by macrophages obtained from C57BL/6 mice, whose stimulation responses may differ from those of BALB/c mice macrophages, yet correspond to their phenotypic characteristics (136) in which extensively reflects the immune signaling, gene regulation, as well as basal functional states. The inherent genetic differences, apart from isolation or culture methods, can significantly affect the polarization capacity, inflammatory signaling, and functional outcomes. In addition, other variables such as mouse age and sex, among other factors, can influence macrophage biology and thereby affect subsequent experimental results (137, 138). Therefore, thorough consideration of these, including strain, tissue origin, and experimental conditions, is a prerequisite for the proper approach to the design of studies and the correct interpretation of their translational relevance in macrophage polarization ​​​​​​research.

### 4.3 Stimulation of macrophage polarization: differences in doses, timing, and potential toxic effects of specific inducers

The optimization of the concentration, duration, and timing of stimuli for macrophage polarization is among the most critical factors, as these directly determine experimental outcomes. As explained previously, from M0 state to the pro-inflammatory M1 phenotype is mostly induced by the cytokine IFN-γ. Also, the role of LPS is important, as the combined with IFN-γ directs macrophages toward M1 phenotype (68). Their synergistic effect is especially relevant in experiments where a strong and distinct M1 macrophage population is required for further functional analyses. Different studies showed variable concentrations of IFN-γ and LPS have been used to induce M1 macrophages either as single agents or combinations. For example, many research works used LPS at 1–100 ng/mL along with IFN-γ at 1–50 ng/mL, and the duration of treatment is 12–24 h that varies according to the study designed and the activation level needed (139, 140). It is also important to note that excessive doses or prolonged exposure to LPS can reduce cell viability and inhibit cell proliferation (141). Overstimulation might lead to exaggerated inflammatory responses which might not be representative of physiological or pathological conditions, and this may complicate the interpretation of the data. Moreover, macrophages pre-primed with a cytokine can also exhibit a more robust polarization upon subsequent stimulation. This priming step prepares macrophage cells with certain factors or environments, thus empowering them to a specific stimulation upon their subsequent activation (142). For example, M1 polarization implies that priming normally means the very first period of exposure to IFN-γ, and then the main inducers such as LPS are being introduced. The previous exposure to IFN-γ makes macrophages more sensitive to subsequent LPS induction, which leads them to an M1 phenotype that is accompanied by the upregulation of the pro-inflammatory response. On the other hand, for anti-inflammatory induction, protocols commonly require macrophage exposure to IL-4 or IL-13 together with 5–40 ng/mL for 14–24 hours or longer (143). Also, IL-10 can be used at the same concentrations to promote solid M2 phenotype (144). The determination of M2 macrophage polarization also depends on cytokine concentration, exposure duration, and their combinations, as these factors influence the outcome; therefore, these should be adjusted according to the experimental setup. Under these treatments, macrophages usually overexpress CD206 and Arg1, have increased IL-10 and TGF-β secretion, and demonstrate enhanced tissue repair-related phagocytic activity. Co-stimulation of macrophages with IL-4 and IL-10 can synergistically enhance anti-inflammatory polarization and immunoregulatory effects; however, they should be consistent in terms of the time to ensure reproducibility. Besides cytokine stimulation, synthetic glucocorticoids (GCs) such as dexamethasone (with 10–^5^ M for 48 h), has potent anti-inflammatory activity, drives macrophage polarization toward an M2 phenotype, characterized by increased expression of markers such as CD163, CD206, and Arg1, while concurrently suppressing the production of proinflammatory mediators including TNF-α and IL-1β (145). Exposure of M2 macrophages to dexamethasone (1mM/mL for 48 h) for 48 hours results in elevated levels of CD163 and CD206, indicative of a phenotypical transition from the M1 to the M2c subtype (146). Additionally, knowing the effects of alternative cytokines on macrophage M1 and M2 polarization, as well as the phenotypic changes, helps to comprehend the mechanistic aspects in different pathologies.

## 5 Quantitative laboratory methods for evaluating macrophage polarization

### 5.1 Marker expression analysis by flow cytometry

Investigation into macrophage polarization involves a broad range of experimental approaches aimed at elucidating the diverse states, functional properties, and phenotypic heterogeneity of macrophages. Accordingly, multiple laboratory techniques are employed for the quantitative and qualitative assessment of macrophage activation states, their functional characteristics, and their interactions with other cellular components as well as molecular signaling pathways within the microenvironment. Among these, flow cytometry (147) is one of the most widely used and powerful techniques for the identification and quantification of macrophage populations based on the expression of specific surface and intracellular markers. This analytical approach enables the discrimination of macrophage subsets based on differential marker expression profiles and provides a quantitative framework for assessing polarization status. Beyond phenotypic classification, the capabilities of flow cytometry extend to functional analyses, including evaluation of cytokine secretion patterns, phagocytic activity, antigen presentation capacity, and expression of activation or signaling-related molecules. As a multiparametric single-cell technique, it allows simultaneous measurement of multiple markers within heterogeneous cell populations, thereby providing a high-resolution view of macrophage diversity. Furthermore, when combined with fluorescence-activated cell sorting (FACS), flow cytometry enables the isolation of highly defined macrophage subpopulations for downstream applications such as transcriptomic profiling, proteomic analysis, and functional assays. This integration of phenotypic identification with cell sorting provides a powerful platform for dissecting macrophage heterogeneity and enables a more comprehensive and mechanistic understanding of macrophage phenotype, functional plasticity, and their role in physiological and pathological processes.

### 5.2 Gene expression profiling in macrophage polarization analysis

Identification​​​​​​ of gene expressions is a primary tool in mechanisms that reveal macrophage dynamics and deepen the understanding of macrophage polarization. This method is used in unmasking the transcriptional programs that illustrate the specific macrophage phenotypes in different pathologies. Along with some restrictions in providing comprehensive and targeted information on surface markers, such techniques as flow cytometry frequently put a limit on the predefined targets, and the main difficulty consists in not detecting the full complexity of transcriptional changes. On the other hand, a study of gene expression allows conducting an unbiased genome-wide search for gene regulation that opens the possibility of finding new transcripts, signaling pathways, and regulatory networks associated with macrophage polarization (148). One of the main techniques is RNA Sequencing (RNA-Seq), which implies the sequencing of the whole RNA content of each macrophage subtype so that their genes expression can be figured out. The technology essentially reveals the prevailing gene expression patterns during macrophage polarization; thus, it can be used to point out the genetic shifts in these cells (149, 150). Also, Quantitative Real-Time PCR (qRT-PCR) is a technique used to amplify and quantify targeted DNA sequences, which is an accurate way to evaluate gene expression and, thus, to identify M1 or M2 macrophage profiles as well as reveal their functions in different physiological and pathological responses (151). This method is a necessary tool in revealing the diversity hidden within the macrophage populations. Additionally, gene​​​​​​ arrays or micro arrays enable high-throughput fixed profiling of a set of genes and provide a fast and relatively cheap way to measure transcriptional changes of multiple samples. This method is effective in assessing macrophage polarization by monitoring the expression of pre-determined sets of M1- or M2-associated genes; however, their effectiveness depends on the probes present on the array (152). NanoString technology is a very sensitive, multiplexed method for the direct quantification of RNA transcripts that does not involve any amplification step that creates cDNA (153). Therefore, it is a suitable method for uncovering low-abundance targets and confirming gene expression patterns related to macrophage polarization, as the detection of subtle changes between M1, M2, and intermediate phenotypes. Besides, other epigenomic methods such as ATAC-seq (Assay for Transposase-Accessible Chromatin with high-throughput sequencing) identifies chromatin accessibility and regulatory elements leading to the inference of transcription factor networks and the revelation of mechanisms governing macrophage identity and activation (150). In combination, these strategies not only support classical gene expression studies but also hold significant potential for macrophage polarization research as they allow the accurate resolution of the dynamic transcriptional and epigenetic shifts that underline M1, M2, and intermediate phenotypes thereby enriching our insight into macrophage function and ​​​​​​plasticity.

### 5.3 Immunostaining approaches and cytokine secretion profiling

On top of that, macrophage polarization studies can benefit from a comprehensive perspective provided by different immunological staining methods, such as immunocytochemistry (ICC) and immunohistochemistry (IHC), which use antibodies to detect specific proteins. ICC is used for proteins in isolated or cultured cells, while IHC allows protein detection within tissue sections, preserving tissue architecture (154). These methods not only identify protein expression but also provide spatial information that is essential for understanding macrophage function and polarization at both the cellular and tissue level (155). For instance, specific markers can be used to identify each macrophage phenotype such as CD86 or MHC-II for M1, and CD206 or Arg1 for M2 macrophages. These visualization techniques are important because they provide a powerful method to demonstrate and differentiate macrophage phenotypes within cells or tissue structures, which is not possible with flow cytometry or bulk RNA analysis. Particularly, macrophage infiltration into tumor microenvironment or chronically inflamed tissues is typically assessed by IHC, meanwhile, ICC and IF are more appropriate for cultured cells or frozen tissue sections, providing higher resolution at the single-cell level (156, 157). Through multiplex fluorescent staining, researchers are able to identify M1 and M2 markers at the same time in a single sample, thus providing a means to evaluate macrophage heterogeneity and plasticity by the quantification of mean fluorescence intensity (MFI). Moreover, cytokine secretion profiling is a fundamental assay often performed using Enzyme-Linked Immunosorbent Assay (ELISA), also multiplex bead-based arrays, or intracellular cytokine staining. Following polarization treatments, supernatants can be collected and analyzed to identify macrophage subtypes based on the unique cytokines they produce (158, 159). Importantly, these profiles not only confirm polarization status but also provide insights into the immunomodulatory roles of macrophages in diseases such as autoimmunity and cancer. For instance, a shift toward an M2-like cytokine profile in the tumor microenvironment frequently correlates with immune suppression and poor prognosis.

### 5.4 Alternative polarization manipulation studies: besides classical M1/M2 activation

**Figure 5:**
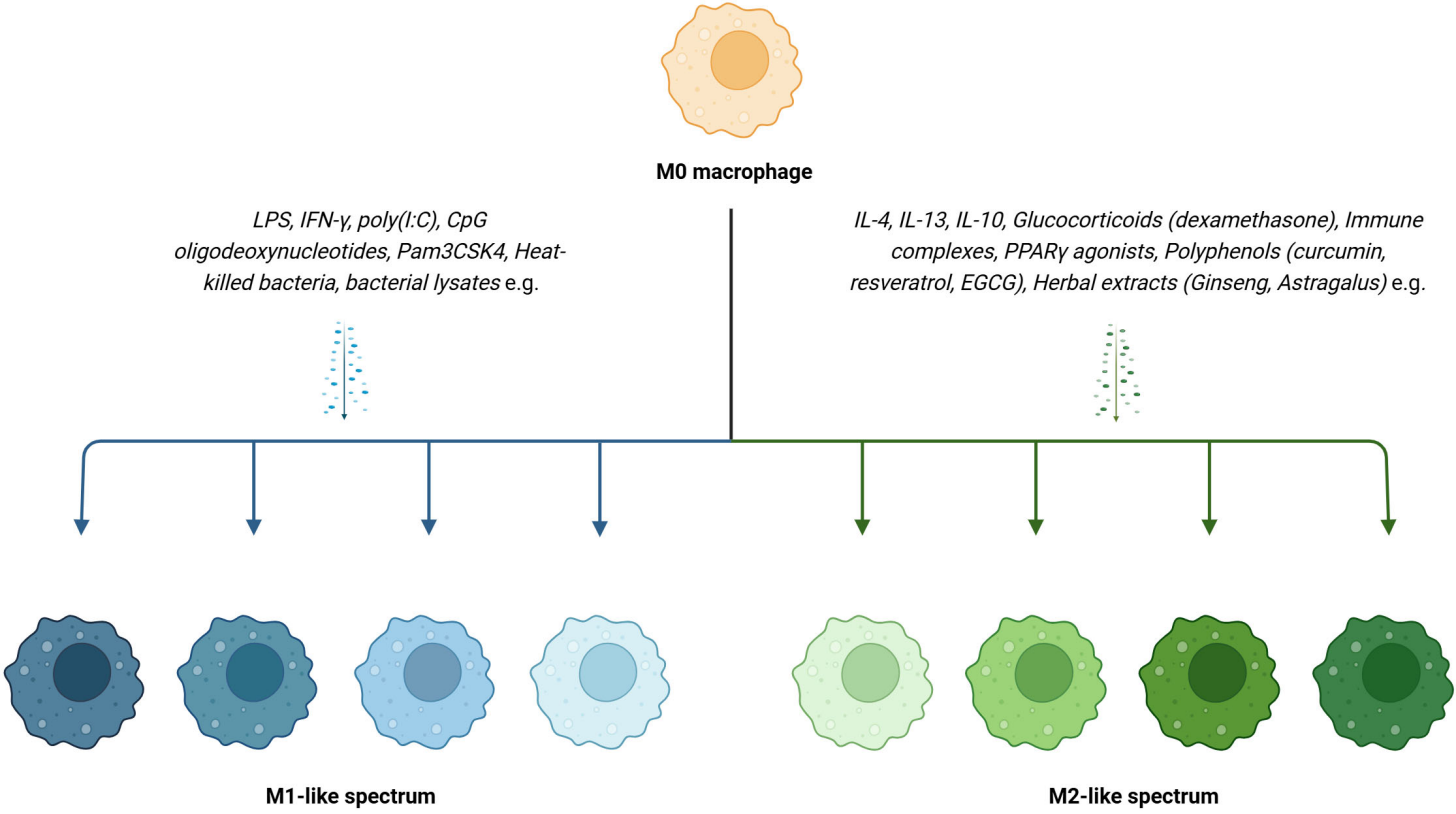
Spectrum of macrophage polarization driven by canonical and alternative stimuli. Macrophages exist on a spectrum of activation states beyond classical M1 and M2. They can be polarized or reprogrammed using diverse stimuli, including pathogen-associated molecular patterns (poly(I:C), CpG ODN, Pam3CSK4, flagellin, heat-killed bacteria) for M1-like phenotypes, and immunomodulatory agents (glucocorticoids, immune complexes, PPARγ agonists, lactate, polyphenols, herbal extracts) for M2-like phenotypes, besides standard macrophage polarization inducers. Figures were created using BioRender (2026).

Recent methodological advances have expanded the range of macrophage polarization inducers beyond classical standard stimuli and have highlighted that macrophage polarization occurs in mixed phenotypic states. Traditionally, macrophage polarization has been categorized as M1 and M2 states induced by standard inducers as explained previously. Beyond these, numerous alternative stimuli have been investigated to refine macrophage polarization models that better mimic physiological or pathological contexts. Macrophage polarization is not binary but exists on a spectrum, shaped by the type and combination of signals in the tissue microenvironment. They​​​​​​ can also change into several different M1-like or M2-like phenotypes, which can be either manipulated or triggered by a wide variety of natural inducers, and not only by the standard inducers as shown in **Figure 5** (14).

Specific combinations of stimuli can be used to induce different M1 or M2 subtypes, each with distinct molecular profiles and functional effects. These experimental techniques are essential for advancing macrophage biology and evaluating their translational potential. PAMPs such as poly(I:C), CpG oligodeoxynucleotides, also Pam3CSK4, and flagellin activate distinct TLRs, promoting M1 inflammatory phenotypes (160). Also, heat-killed bacteria can also promote classical activation. Conversely, immunomodulatory agents including colony-immune complex, PPARγ agonists, and metabolic cues such as lactate induce M2-like states supporting immunoregulation, tissue remodeling, and wound repair (161). These noncanonical inducers enhance our understanding of macrophage heterogeneity and enable modeling of disease-specific phenotypes such as tumor-associated or infection-associated macrophages both *in vitro* and *in vivo*. In​​​​​​ addition, other studies showed that nanoparticle-based technologies aim at the direct delivery of immunomodulatory agents to macrophages. These agents may be cytokines, TLR agonists, or small molecules, and therefore, M1 or M2 polarization can be induced in a regulated way (162). Micro or nano scaled particles, PLGA, liposomes, or gold nanoparticles, functionalized with the proper ligands, make the targeted delivery to tissue-resident macrophages or TAMs possible, thus lessening the side-effects and, at the same time, elevating the efficiency of the reprogramming process (163)​​​​​. Moreover, plant-based compounds and natural products, which broadly include polyphenols (curcumin, resveratrol, EGCG), alkaloids, and powerful natural ingredients such as Ginseng or Astragalus. These substances were inductively identified to affect the macrophage-associated signaling pathways such as NF-κB, STAT, and MAPK and as a result change the pro- or anti-inflammatory characteristics of the cells, depending on the given situation (164, 165). Additionally, gene-based interventions provide a far more precise way of controlling macrophage polarization: the overexpression or knockdown of transcription factors, CRISPR/Cas9-mediated gene editing, or the administration of specific microRNAs (miR-155 for M1, miR-223 for M2) can help macrophages achieve the desired phenotypes (166).

## 6 Conclusion and discussion

The macrophages, as highly plastic and heterogeneous immune cells, continuously integrate microenvironmental signals and dynamically adapt their functional programs to maintain physiological balance or respond to injury and infection. The classical M1/M2 polarization framework has provided a useful conceptual foundation for understanding macrophage functional diversity, particularly in controlled experimental systems. However, the many evidence demonstrates that macrophage phenotypes *in vivo* extend far beyond its binary classification, existing instead along a dynamic and context-dependent spectrum shaped by cytokines, metabolic cues, tissue-specific factors, and temporal changes within the microenvironment. Hence, throughout this review, we have discussed the molecular pathways and biomarkers associated with M1 and M2 polarization, emphasizing key signaling networks, surface markers, their cytokine release profiles, and functional characteristics that define pro-inflammatory and anti-inflammatory states. While these markers remain valuable tools, their interpretation requires caution due to overlapping expression patterns and the inherent plasticity of macrophages. Simply, no single marker is sufficient to define macrophage identity, and multiparametric and integrative approaches are necessary to achieve reliable characterization. We have also highlighted the importance of macrophage polarization in diverse pathological contexts, including infectious diseases, chronic inflammatory and autoimmune disorders, cancer, fibrosis, atherosclerosis, and tissue repair. In many disease conditions, dysregulated macrophage activation contributes directly to disease progression. Conversely, modulation of macrophage phenotype holds considerable therapeutic or diagnostic promise. Strategies aimed at reprogramming macrophages, rather than simply suppressing inflammation responses, may offer more precise and durable clinical outcomes. Moreover, methodological considerations in macrophage polarization research are crucial (*see* **Table 4**). Concepts including variability in cell sources, stimulation protocols, cytokine doses, timing, animal models, and analytical methods significantly affect experimental outcomes and reproducibility. Standardization of polarization protocols, careful validation of markers, and transparent reporting of experimental conditions are essential to improve comparability across studies. Thus, emerging technologies such as high-dimensional flow cytometry, transcriptomic profiling, and real-time functional assays provide powerful tools to dissect macrophage heterogeneity with greater resolution, yet they must be applied within well-controlled and reproducible experimental frameworks. Also, this field is moving toward a more nuanced understanding of macrophage biology that integrates metabolic programming, epigenetic regulation, spatial organization within tissues, and dynamic repolarization capacity. Recognizing macrophage polarization as a flexible and reversible process rather than a fixed state opens new avenues for therapeutic intervention. Future research should prioritize longitudinal and *in vivo* analyses to better capture the dynamic transitions of macrophages across disease stages. Hence, advancing our understanding of macrophage polarization requires both conceptual refinement beyond the classical M1/M2 paradigm and methodological rigor in experimental design, using standardized protocols and high-resolution analytical approaches to move toward a more precise definition of macrophage functional states. Such progress will be critical for translating basic macrophage biology into reliable biomarkers and innovative therapeutic strategies across a wide spectrum of inflammatory, infectious, and other chronic diseases.

**Table 4:** Comparison of *common* methods for different research purposes.

#	Common methods	Specificity; key readouts	Research purpose; applications	Ref.
1	Flow Cytometry	Macrophage surface markers, intracellular cytokines; other related proteins	Quantitative assessment of M0-M2 populations; functional characterization; cell sorting for downstream analyses; rapid, multiparametric analysis	(147)
2	Cytokine Profiling (ELISA, Multiplex Bead Arrays, Intracellular Staining)	Secreted or intracellular cytokines	Confirms polarization status; assesses immunomodulatory function; evaluates responses in disease models	(159)
3	Quantitative Real-Time PCR (qRT-PCR)	Targeted gene expression of specific M1/M2 markers	Accurate quantification of selected genes; validation of polarization markers; functional assessment of macrophage subtypes	(151)
4	RNA Sequencing (RNA-Seq, scRNA-Seq)	Genome-wide transcriptional signatures; single-cell resolution for heterogeneous populations	Unbiased identification of polarization-specific genes; discovery of novel transcripts and regulatory networks; high-resolution analysis of heterogeneous populations; aids discovery of therapeutic targets	(167)
5	Immunostaining (ICC, IHC)	Spatial localization and expression of specific markers (mean fluorescence intensity quantification	Visualizes macrophage phenotypes in tissues or cultured cells; assesses spatial heterogeneity and plasticity; allows live-cell imaging for dynamic studies	(155)
